# Ductile Fracture of L360QS Pipeline Steel Under Multi-Axial Stress States

**DOI:** 10.3390/ma18245582

**Published:** 2025-12-12

**Authors:** Hong Zheng, Bin Jia, Li Zhu, Naixian Li, Youcai Xiang, Jianfeng Lu, Shiqi Zhang

**Affiliations:** 1 School of Civil Engineering and Architecture, Southwest University of Science and Technology, Mianyang 621010, China; zhengh1106@163.com (H.Z.); linaixian23@gscaep.ac.cn (N.L.); xyc113579@126.com (Y.X.); 2 Deyuan Technology Co., Ltd., Chengdu 610095, China

**Keywords:** L360QS pipeline steel, ductile fracture, finite element analysis, post-necking behavior, stress triaxiality

## Abstract

L360QS pipeline steel, due to its high toughness, high strength, resistance to sulfide stress cracking, and resistance to hydrogen-induced cracking, is increasingly being used in pipeline network construction. Its fracture behavior is a critical factor for safe operation in mountainous steep-slope environments, but it has not yet been widely studied. Therefore, this paper conducts extensive experiments on the ductile fracture of L360QS pipeline steel. The tests employed standard tensile, notched tensile, shear, and compression specimens, covering a stress triaxiality range from approximately −0.33 to 0.92. The study combined Ling’s iterative method to establish an elastoplastic constitutive model considering post-necking behavior, and incorporated it into finite element models to extract the average stress triaxiality and equivalent plastic strain at the moment of fracture initiation for each type of specimen. Based on the extracted data, a piecewise ductile fracture model was established: a simplified Johnson–Cook criterion is used in the high triaxiality range, while an empirical function is used to describe fracture behavior in the medium, low, and negative triaxiality ranges. The model was validated using a train–test split approach, predicting fracture displacements for an independent test set of specimens. The results showed all prediction errors were within 5%, demonstrating the model’s high accuracy. Furthermore, a Spearman correlation analysis quantified the influence of geometric factors, revealing that notch curvature has the strongest monotonic relationship in controlling average stress triaxiality and fracture strain. The fracture model established in this paper can accurately predict the fracture behavior of L360QS pipeline steel and provides a reliable basis for failure prediction and safety assessment under complex service conditions (such as mountainous steep slopes).

## 1. Introduction

Global demand for clean energy and the transition of the energy structure are driving oil and gas exploration into extreme environments such as deep sea, polar regions, and mountainous steep slopes [1,2]. At the same time, using existing pipelines to transport hydrogen or hydrogen-blended natural gas has become a key route to achieve the “carbon neutrality” goal [3,4]. This places higher performance requirements on the service safety of pipeline materials, especially requiring materials to have excellent resistance to hydrogen-induced cracking (HIC) [5,6] and sulfide stress cracking (SCC) [7,8]. L360QS pipeline steel, with its high strength, high toughness, excellent weldability, and outstanding HIC/SCC resistance, has become the preferred material for pipeline construction in high-sulfur oil and gas fields [9,10]. However, during service, pipelines inevitably develop defects such as dents and scratches due to geological hazards, third-party damage, or corrosion [11]. Under complex stress conditions, these defects can easily initiate cracks, eventually leading to ductile fracture [12]. Therefore, systematically studying the ductile fracture characteristics and failure mechanisms of L360QS steel is the physical basis and key prerequisite for implementing pipeline safety operation and maintenance strategies based on fitness-for-service assessments (e.g., API 579-1/ASME FFS-1 [13] and BS 7910 [14]).

The ductile fracture of metals is essentially a complex damage evolution process involving the nucleation, growth, and coalescence of micro-voids [15,16]. At the microscale (i.e., the void mechanism level), McClintock [17] and Rice & Tracey [18] revealed the exponential effect of hydrostatic pressure (i.e., stress triaxiality) on void growth, laying the theoretical foundation for the central role of stress state in ductile fracture. High stress triaxiality greatly promotes spherical void growth, thereby sharply reducing the material’s plastic limit and thus its toughness [19]. Because the tips of defects (such as dents, scratches) in pressurized structures like pipelines are typical high-triaxiality regions, accurately predicting material fracture behavior under this constrained state is key to structural safety assessment. Subsequently, Gurson [20], Tvergaard [21,22] and Needleman [23] established the classic GTN damage model based on these physical mechanisms. However, due to the complexity of calibrating GTN model parameters [24], macroscopic phenomenological fracture models based on equivalent plastic strain are more favored in engineering for their efficiency. Early macroscopic models, such as the classical criterion proposed by Johnson and Cook [25], were developed to characterize the highly dependent exponential decline of fracture strain with increasing stress triaxiality in the high-triaxiality range. However, Bao and Wierzbicki [26] further found that the relationship between fracture strain and stress triaxiality is not monotonically decreasing; there is a “valley” of fracture toughness in the low triaxiality (shear-dominated) range. This finding proved that stress state cannot be fully described by triaxiality alone and that a Lode angle parameter (θ) or normalized third deviatoric stress invariant must be introduced [27]. Based on this, Bai and Wierzbicki [28,29] proposed the Modified Mohr–Coulomb (MMC) criterion, establishing a three-dimensional fracture locus. Subsequently, a series of models, such as the Hosford–Coulomb (HC) model [27,30], improved the prediction accuracy in the medium- and low-triaxiality ranges [31]. However, the J–C model was not invalidated, but rather precisely identified as representing an axisymmetric tension stress state with a normalized Lode angle parameter of θ = 1 (according to the convention in Ref.) [32]. This corresponds to the dominant failure mode of local high-constraint defects in pipelines caused by pitting [33] or mechanical dents [34], which can be simplified by the axisymmetric principle. Therefore, J–C model parameters remain the most direct and efficient approach for solving such engineering problems.

On this basis, many researchers have conducted extensive studies on different steel grades. For example, Oh et al. [35] for X65 steel developed a modified fracture strain model considering stress state; Fagnana et al. [36] and Zhang et al. [37] conducted systematic ductile fracture analyses and calibrated GTN models for X70 steel; Min et al. [38] further established a combined fracture criterion for X70 steel under combined tension-shear stress states. For X80 steel, Liu et al. [39] and Papasidero et al. [40] compared the applicability of various fracture models, and Rakin & Sedmak [41] extended the research to ductile fracture initiation in high-strength steel welded joints. As the steel grade increases, Gao & Wierzbicki [42] applied fracture models to X100 steel. This series of in-depth studies on high-grade pipeline steels highlights the critical importance of fracture parameters for ensuring safety assessments. However, L360QS differs fundamentally from these high-strength grades (X70/X80/X100) in its microstructural design, which prioritizes resistance to Sulfide Stress Cracking (SSC) over absolute strength [9,10]. The fracture locus of such SSC-resistant microstructures under complex constraints has not been systematically mapped. Relying on fracture parameters derived from standard high-strength steels could lead to inaccurate assessments for L360QS pipelines operating in geologically unstable regions. Current research on L360QS is predominantly focused on electrochemical corrosion mechanisms and uniaxial tensile properties. There is a critical absence of a calibrated phenomenological failure model that covers the wide range of stress triaxialities encountered during geohazard-induced buckling or denting. This study aims to fill this specific gap by constructing a comprehensive fracture locus, enabling more accurate strain-based design and assessment. This gap results in insufficient test data and accurate failure models to support safety evaluations of its service performance in complex stress environments like mountainous steep slopes.

Based on this, the present study conducts a systematic investigation of the fracture characteristics of L360QS pipeline steel under multi-axial stress states. First, through fracture experiments under multi-axial stress states, load–displacement curves of L360QS were obtained over different stress triaxiality ranges (from −0.33 to 0.92). Then, Ling’s iterative method [43] was combined to establish a true stress–strain constitutive model. Finite element simulations were used to extract the average stress triaxiality and fracture strain of the critical element at fracture initiation for each specimen. On this basis, a piecewise failure model was developed: a simplified Johnson–Cook model is used in the high stress triaxiality range, while a specific function form is fitted for the medium, low, and negative triaxiality ranges. To verify the model’s reliability, a “train–test split” method [44] was employed, and the fracture displacements of test set specimens were used to validate the model. Finally, Spearman’s rank correlation coefficient [45] was introduced to quantitatively analyze the strong monotonic relationships between notch curvature, stress state, and fracture strain. The failure model constructed in this study can provide critical material data and model support for the integrity assessment and fracture risk analysis of in-service pipelines under complex conditions such as mountainous steep slopes.

## 2. Materials and Methods

### 2.1. Material and Experimental Program

**Figure 1:**
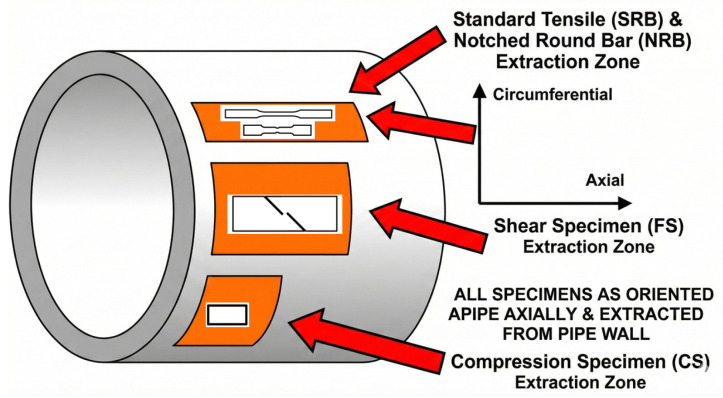
Schematic of specimen sampling.

All specimen material was taken from the axial direction of an in-service L360QS natural gas pipeline (outer diameter 406.4 mm, wall thickness 12.5 mm), as shown schematically in Figure 1 and Table 1.

**Figure 2:**
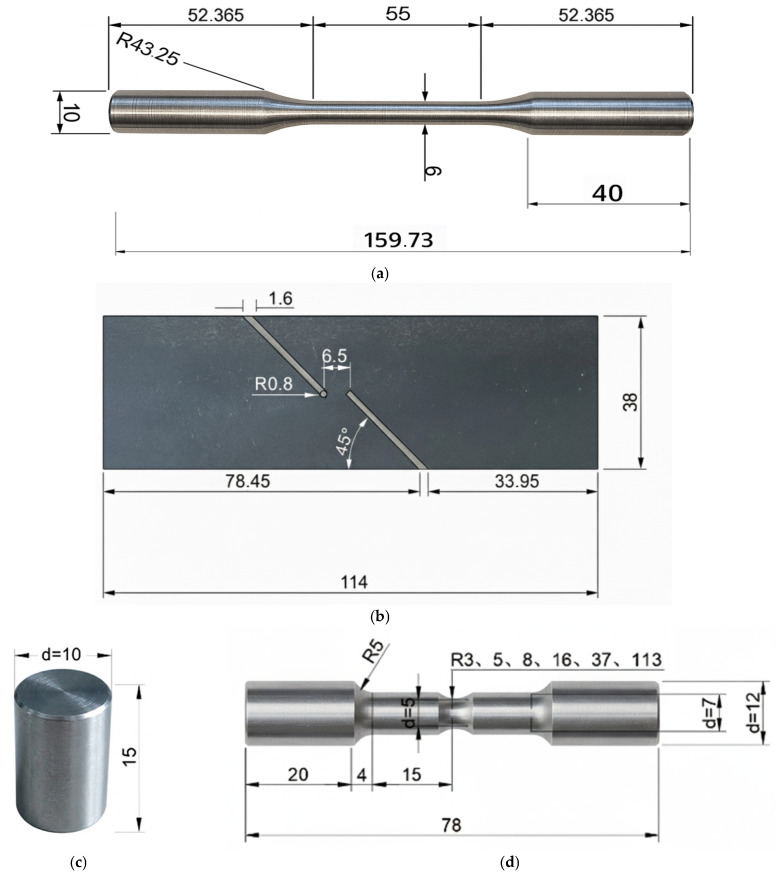
Geometries of the test specimens (dimensions in mm): (**a**) Standard tensile specimen (SRB); (**b**) Shear specimen (FS, thickness 12.5 mm); (**c**) Compression specimen (CS); (**d**) Notched round bar (NRB) specimen.

To characterize the material’s ductile fracture behavior under various stress states, nine specimen geometries were designed as illustrated in Figure 2, with five repetitions performed for each type to ensure reliability. These geometries include standard tensile specimens (SRB) prepared according to GB/T 228.1-2021 [46] to determine fundamental constitutive responses; six notched round bar (NRB) specimens (R = 3, 5, 8, 16, 37, and 113 mm) based on HB 5214-1996 [47] to systematically vary stress constraints; a flat-grooved shear specimen (FS) fabricated per HB 6736-1993 [48] to represent low-triaxiality conditions while maintaining the 12.5 mm pipe wall thickness; and a cylindrical compression specimen (CS) designed according to GB/T 7314-2017 [49] to characterize behavior under negative stress triaxiality.

These nine different types of specimens were all sampled in the axial direction of the pipe. The shear specimen is used to determine the fracture behavior at low stress triaxiality (with the expected triaxiality near 0 at the notch). The compression specimen is used to determine fracture behavior at negative stress triaxiality (expected triaxiality around –0.333). The notched specimens have notches with radii of 3 mm, 5 mm, 8 mm, 16 mm, 37 mm, and 113 mm, whose corresponding initial stress triaxialities can be estimated by Formula (2); roughly, the stress triaxialities are 0.826, 0.649, 0.538, 0.439, 0.380, 0.349, respectively.

**Figure 3:**
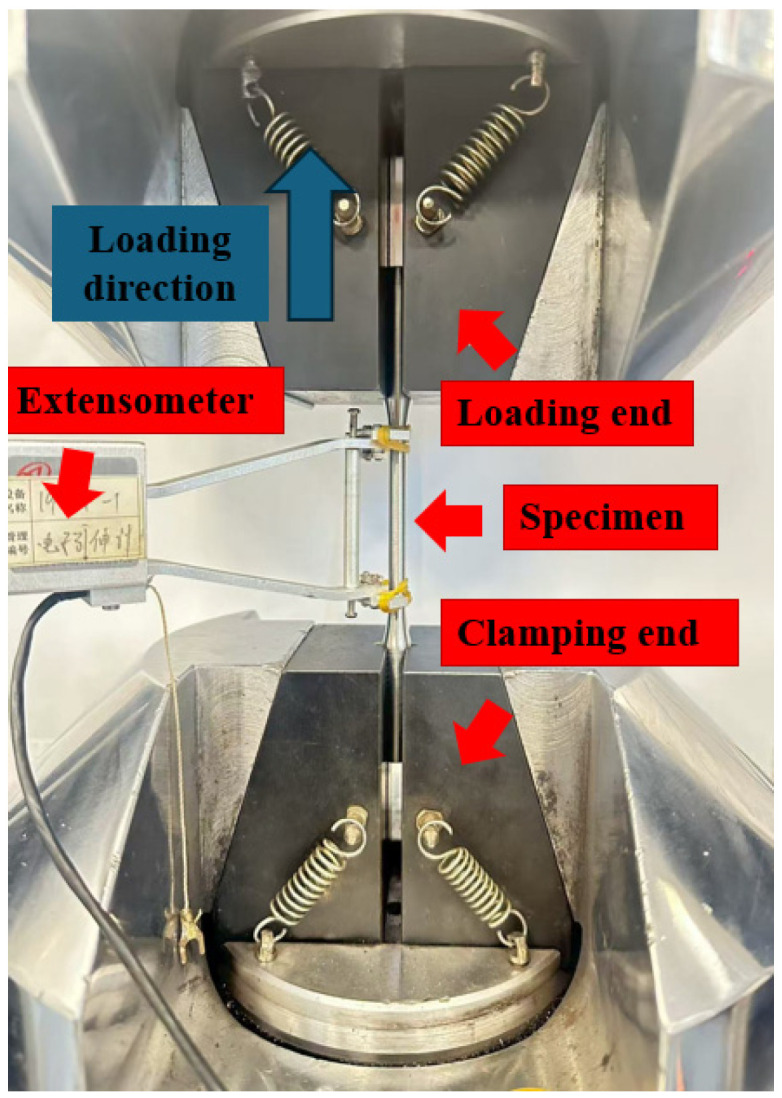
Test procedure.

All mechanical tests were performed on a CMT5150 electronic universal testing machine (Meister Industrial System Co., Ltd., Shenzhen, China). The machine is equipped with a load cell with a maximum capacity of 100 kN, achieving a force measurement accuracy of Class 0.5 (indication error within ±0.5%) according to ISO 7500-1 [50]. Displacement was measured using a high-precision extensometer, with an accuracy class meeting ASTM E83 Class B-1 standards [51], ensuring the accuracy of elastic modulus measurement in the small deformation stage. The equipment was calibrated according to national metrology standards prior to experimentation, as shown schematically in Figure 3. Tests were conducted at room temperature with a quasi-static loading rate of 0.45 mm/min.

### 2.2. Stress State Characterization

Stress state, specifically stress triaxiality, has been proven to be the most important factor affecting fracture strain. Stress triaxiality (η) is a nondimensional parameter expressed as the ratio between hydrostatic pressure and the Mises equivalent stress [26]:

(1) $$\eta =\frac{\sigma_{m}}{\sigma_{e}}=\frac{I_{1}/3}{\sqrt{3J_{2}}}$$

where $\sigma_{m}$ is the hydrostatic pressure (mean stress); $\sigma_{e}$ is the Mises equivalent stress; $I_{1}$ is the first invariant of the stress tensor, representing volumetric effect; $J_{2}$ is the second invariant of the deviatoric stress tensor, representing shape change effect (plastic deformation).

The physical meaning of η is to quantify the proportion of tension/compression versus shear. For example: uniaxial compression: η=−1/3 (inhibits void growth); pure shear: η=0; uniaxial tension: η=1/3 (reference state); notched tension: η>1/3 (high constraint, promotes fracture).

When establishing a fracture model, η is not a constant value. This is because during a specimen’s plastic deformation (especially in notched specimens), the internal stress state (particularly at the center) is continuously evolving due to geometric nonlinearity from necking, and fracture is the cumulative result of the entire plastic deformation history.

In Bridgman’s classical solution, the initial triaxiality ($\eta_{init}$) can only be estimated from the specimen’s initial geometry to approximate the stress triaxiality at the start of loading [52]:

(2) $$\eta =\frac{1}{3}+\sqrt{2}\mathrm{In}\left(1+\frac{\alpha }{4R}\right)$$

where α is the radius of the notched section; *R* is the notch radius.

This initial value cannot reflect the drastic changes during subsequent deformation, and thus cannot accurately represent the stress state over the entire fracture process. Therefore, this study adopts an experimental–FEM method by introducing the average stress triaxiality ($\eta_{avg}$). $\eta_{avg}$ is defined as the integrated average of stress triaxiality over the entire equivalent plastic strain history (from yield to fracture) [26]:

(3) $$\eta_{avg}=\frac{1}{\varepsilon_{f}}\int_{0}^{\varepsilon_{f}}\eta (\varepsilon_{p})d\varepsilon_{p}$$

where $\eta_{avg}$ is the stress triaxiality; $\varepsilon_{f}$ is the fracture strain; $\varepsilon_{p}$ is the equivalent plastic strain.

$\eta_{avg}$ cannot be directly measured by experiments and must be extracted from finite element (FEM) results. By considering the entire deformation process, it better represents the overall stress state. Through a series of experiments and FEM simulations, The calibrated fracture locus of L360QS pipeline steel was established and formulated as:

(4) $$\varepsilon_{f}=f(\eta_{avg})$$

where $\varepsilon_{f}$ is the fracture strain; $\eta_{avg}$ is the average stress triaxiality.

This function defines the material’s failure criterion: for a given average triaxiality ($\eta_{avg}$), failure is assumed to occur when the local equivalent plastic strain reaches the critical fracture strain corresponding to that $\eta_{avg}$.

### 2.3. Constitutive Model Development and FEM Setup

An accurate elastoplastic constitutive model is required for the FEM simulations. The basic mechanical properties were first determined from the SRB tests.

Traditional engineering stress is based on the initial geometry. Once the material enters plastic deformation, due to cross-sectional contraction, the engineering stress will severely underestimate the true stress actually borne by the material. This deviation causes the engineering stress–strain curve to display a descending segment after reaching the tensile strength, which is not true material softening but a geometric effect caused by necking (plastic instability). If this curve were used to construct a constitutive model, it would lead to misjudgment of the material’s plastic hardening characteristics. Therefore, this study uses a true stress–true strain framework. Assuming constant volume during the pre-necking plastic deformation, conversion is performed via Formulas (1)and (2) [53]. The elastic modulus was derived through linear regression of the elastic region, and the transformation equations were employed up to the uniform deformation stage (before necking) to establish the true stress–strain behavior of L360QS pipeline steel:

(5) $$\sigma_{\mathrm{true}}=\frac{FL}{A_{0}L_{0}}$$

(6) $$\varepsilon_{\mathrm{true}}=\ln\left(\frac{L}{L_{0}}\right)$$

where *F* is the load at the current time; *L* is the specimen’s current length; *L*_0_ is the specimen’s initial length; *A*_0_ is the specimen’s initial cross-sectional area.

When necking occurs in the specimen’s gauge section, the stress and strain in the necked region become non-uniformly distributed. The true stress–strain formulation described above does not hold once necking begins. Consequently, the post-necking stress–strain relationship was established using Ling’s iterative approach [43].

A weighted average method for correcting the post-necking true stress–strain relationship was proposed by Ling [43] in 1996, as expressed in Equation (7). He suggested that the upper bound of the post-necking true stress–strain relationship is a linear relationship given by Formula (8), and the lower bound is a power function relationship given by Formula (9) [54]. The core procedure is:

Using the measured true stress–strain data, fit an initial set of constitutive parameters (i.e., an initial *W* value). Then input this set of parameters into a numerical model of the standard tensile specimen and perform an elastoplastic finite element simulation to extract a simulated load–displacement curve. Next, quantify the deviation between the simulated curve and the experimental curve, and iterate repeatedly until the correlation between the simulation results and test data meets a convergence criterion (e.g., RMSE ≤ 5%).

(7) $$\sigma_{t}=(W)(\alpha \varepsilon_{t}+b)+(1-W)(K\varepsilon_{t}^{n})$$

(8) $$\sigma_{l}=\alpha \varepsilon_{t}+b$$

(9) $$\sigma_{p}=K\varepsilon_{t}^{n}$$

From the stress continuity condition at the necking point and the initial conditions at the onset of necking, one can obtain $\alpha =\sigma_{u}$, $n=\varepsilon_{u}$, b=α(1−n), $K=\alpha /n^{n}$, $\sigma_{u}$ and $\varepsilon_{u}$ are the true stress and true strain at the onset of necking.

**Figure 4:**
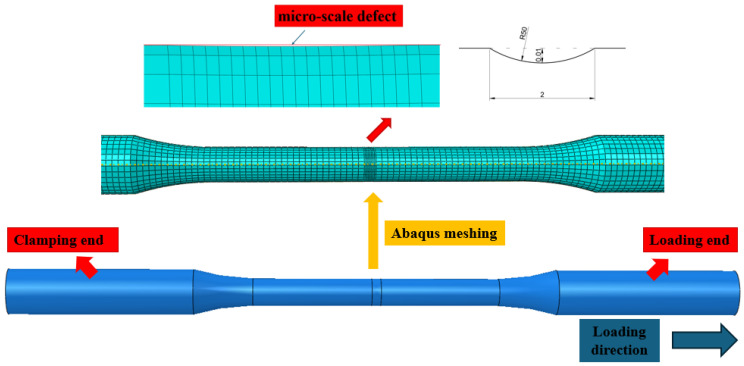
Tensile model of round bar specimen.

ABAQUS software (version 2023) was used to model the specimens at a 1:1 scale. Eight-node C3D8R solid elements were selected. To induce necking at the specimen’s midsection, a 2 mm-long and 0.01 mm-deep arc indentation with a radius of 50 mm was introduced at the center. Local mesh refinement was performed around the indentation, with one end of the specimen constrained and a displacement boundary condition applied at the opposite end, as illustrated in Figure 4. Introducing an artificial indentation in the tensile specimen (note: the schematic dimensions in the figure are not drawn to standard scale, but for illustrative purposes) is a commonly used method in numerical simulation to induce necking [55,56].

Material properties: The elastic parameters of the material use the data from Table 2 above, and the plastic hardening behavior uses the true stress–strain model determined earlier. The yield condition uses the classical Mises yield criterion, and no damage is introduced in the parameter-calibration FE model (pure elastoplastic model).

Boundary conditions: In the experimental process, the specimen’s grip section was completely clamped, so in the model, one end of the specimen is fixed. Specifically, one end of the model has its displacement constrained in the axial direction, and a displacement load is applied in the axial direction at the other end to simulate the tensile behavior of the specimen.

Computation method: To simulate the quasi-static tensile process and handle convergence issues caused by contact and large deformations, the Implicit Dynamic Solver was employed. The non-linear geometry switch was activated. The total simulation time was set to 1 s. An automatic time stepping algorithm was used with an initial increment of 0.001. To ensure the quasi-static nature of the results, the system’s kinetic energy was monitored throughout to ensure it remained below 5% of the internal energy. No artificial damping, mass scaling, or other additional source terms were introduced. We also did not need to apply any under-relaxation factors, as the solution converged with the default solver settings.

Mesh size: A mesh sensitivity analysis was conducted using element sizes of 0.5 mm, 0.2 mm, and 0.1 mm in the notch region.

## 3. Results

### 3.1. Experimental Results

**Figure 5:**
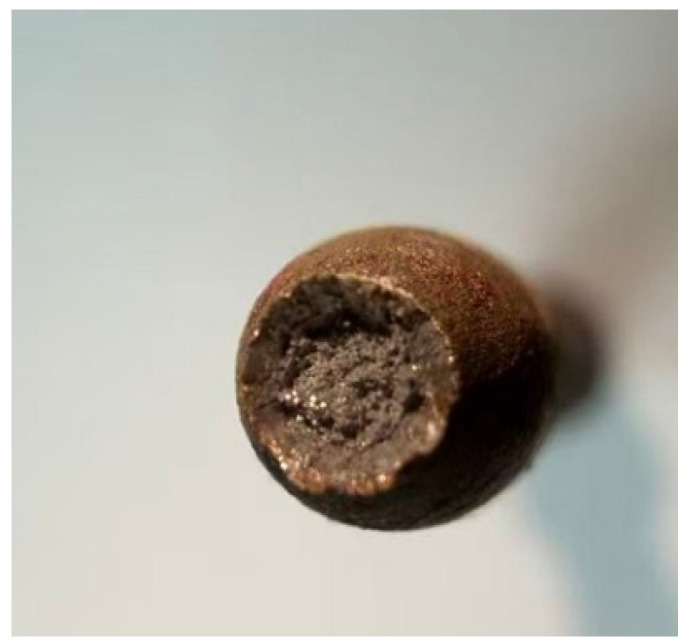
Standard tensile specimens after fracture.

After the tensile specimen fractured, its cross-section appeared as a concave elliptical shape with significant necking. The fracture surface exhibited a rough, fibrous morphology where macroscopic void coalescence features were observed, clearly indicating a ductile failure mechanism characterized by micro-void nucleation and growth, as shown in Figure 5.

**Figure 6:**
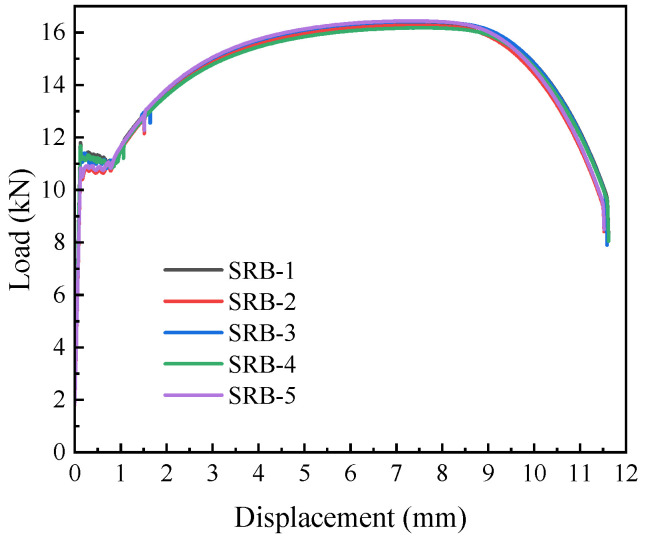
Displacement-load curve of L360QS pipeline steel.

As illustrated in Figure 6, the load–displacement curves of L360QS steel display a noticeable yield plateau, and the close agreement among specimens demonstrates the dependability of the results.

### 3.2. Constitutive Model of L360QS Steel

**Figure 7:**
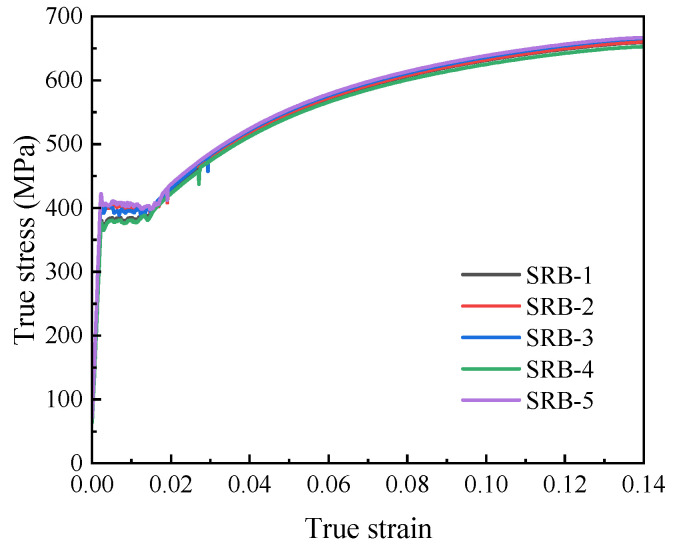
True stress–strain curve of L360QS pipeline steel neck before contraction.

As shown in Figure 7, the true stress–strain curve of L360QS steel before necking was obtained based on the displacement–load response. The mechanical parameters averaged from five repetitions are provided in Table 2.

**Figure 8:**
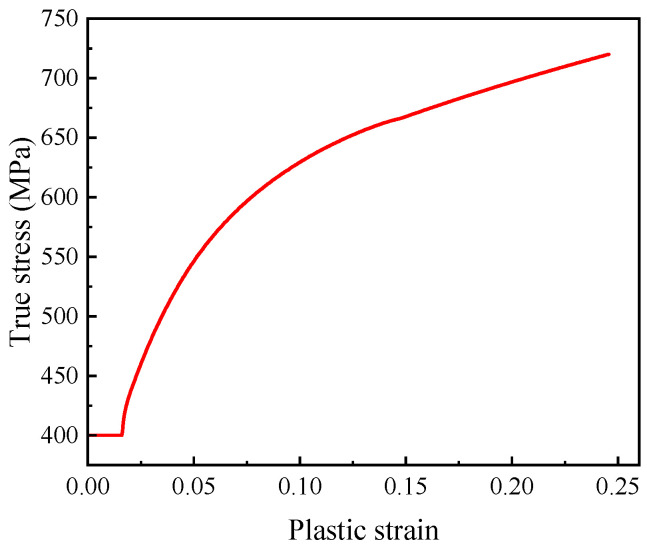
Hardening curve for L360QS pipeline steel.

As *W* increases, the post-necking true stress of L360QS pipeline steel increases, and as plastic strain increases, the divergence between the simulated true stress curve and the experimental true stress curve grows larger. After multiple iterations, it was finally determined that when *W* = 0.32, the analyzed engineering stress matched the experimental results well. As shown in Figure 8, the true stress–plastic strain behavior of L360QS pipeline steel was obtained by applying the true stress–strain formulation prior to necking and Ling’s correction method beyond necking.

**Figure 9:**
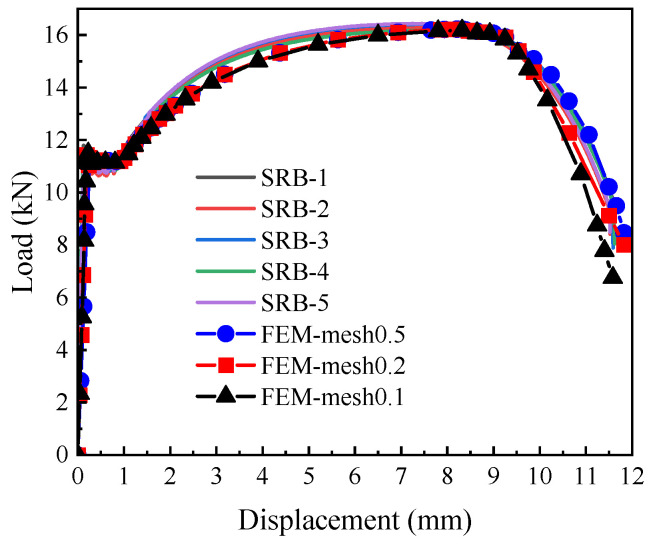
Comparison of constitutive model FEM simulation and tensile test data for L360QS pipeline steel (load–displacement curves).

For L360QS pipeline steel, when W = 0.32, the simulated load matched the experimental results well; the corresponding load–displacement relationship is shown in Figure 9. The slight differences in the experimental specimen are caused by the material’s inherent microscopic inhomogeneity and the systematic error of the testing equipment. The good agreement between the FEM results (showing an RMSE of less than 2%) and the test results indicates that the established true stress–strain model is accurate. The cold expansion process of L360QS pipe manufacturing resulted in material anisotropy, while FEM model used an isotropic assumption. This simplification in constitutive description is the main reason for the slight separation between the simulated and experimental curves in the high strain region.

The results showed that the load and displacement values converged at a mesh size of 0.2 mm, with a difference of less than 2% compared to the 0.1 mm mesh. Consequently, a characteristic element size of 0.2 mm was adopted for all critical regions.

### 3.3. Calibration of Fracture Parameters

Fracture Strain: For round bar specimens, the fracture strain can be calculated by the formula [52]:

(10) $${\bar{\varepsilon }}_{f}=2\ln(\frac{d_{0}}{d_{t}})$$

where d_t_ is the diameter of the fracture cross-section; d_0_ is the specimen’s initial diameter. Due to the experimental difficulty in accurately determining the cross-sectional diameter at the time of fracture and the fact that the final fracture surface is irregular rather than circular, a universally accepted definition of fracture initiation based on fracture strain is not yet available. For finite element simulations, the equivalent plastic strain at fracture can be readily determined from the computational results.

Stress Triaxiality: For identifying stress triaxiality, the initial stress triaxiality was mentioned in Section 2.2, but since it is not representative, the average stress triaxiality was chosen.

**Figure 10:**
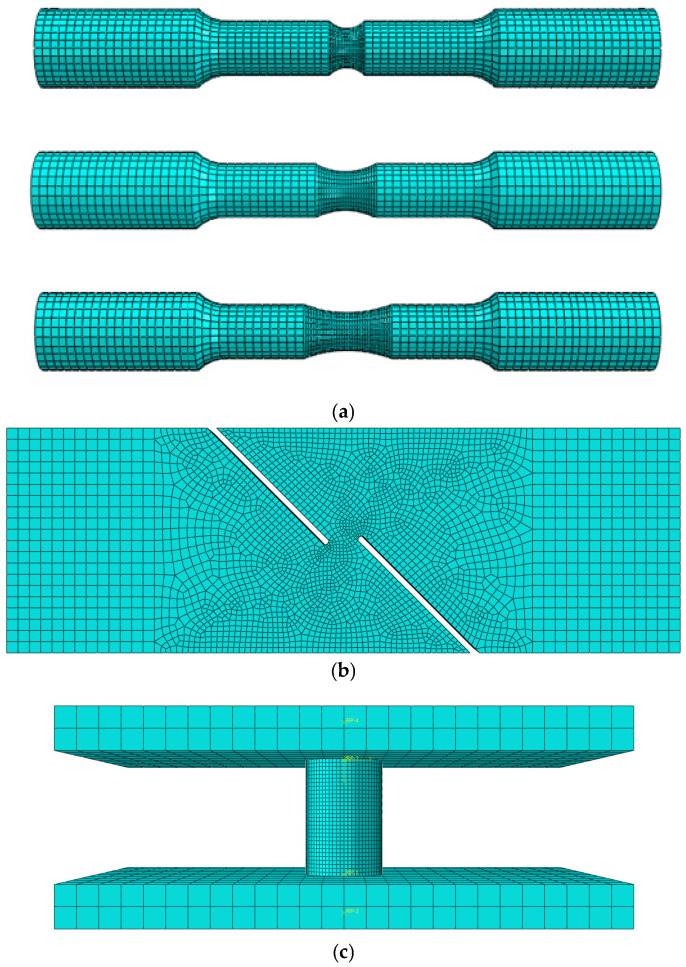
Finite element models of various test specimens: (**a**) NRB3, NRB8, NRB16; (**b**) FS; (**c**) CS.

To obtain the fracture strain and average stress triaxiality at fracture for the specimens, numerical simulations were carried out for the tensile and compression tests of NRB3, NRB8, NRB16, SRB, and FS. Similar to the smooth round bar tensile test, the specimens were modeled 1:1 in ABAQUS. Notably, the middle portion of the shear model remained the pipeline’s original thickness, with a slight arc, which was taken into account in the finite element modeling process. Throughout the experimental procedure, the grip section of the specimen was fully clamped, so in the model, one end was set as fixed—i.e., one end of the model had displacement constraints in the specimen’s length direction—and a displacement was applied at the other end to simulate tensile loading. Eight-node C3D8R solid elements were still used. An implicit dynamic solver was used to simulate the quasi-static loading, keeping the ratio of kinetic energy to internal energy within 5%. For the NRB3, NRB8, NRB16, SRB, and FS models, a local mesh refinement was applied at the notch, as shown in Figure 10:

Because all material properties were assigned only elastic–plastic behavior, the models did not exhibit fracture during the simulation. The average experimental fracture displacement was taken as the onset of cracking, and this parameter was used to align the FEM predictions with the measured load–displacement curves.

**Figure 11:**
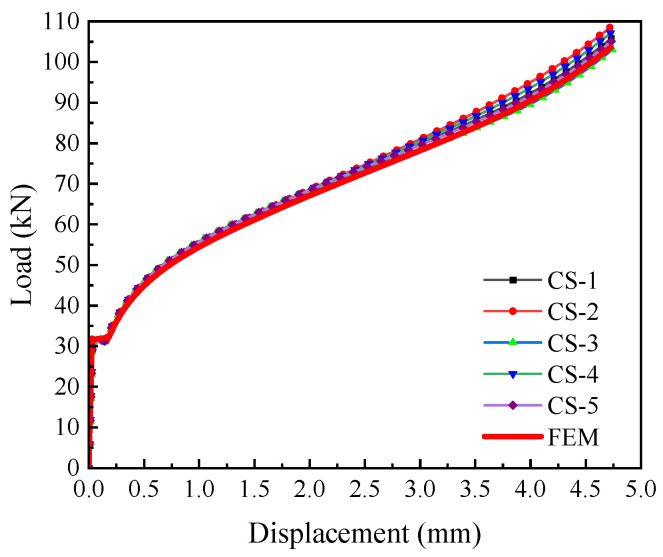
Comparison of finite element and compression tests.

For the compression specimen, since it endured high compressive stress and no cracking or fracture occurred during the test, the compression test data and simulation results were only used to verify the constitutive model’s accuracy in the negative triaxiality range, and not used to calibrate failure parameters. As shown in Figure 11, the numerical simulation matches the experimental results well, proving that the constitutive model established in this study is quite accurate in the negative triaxiality range.

**Figure 12:**
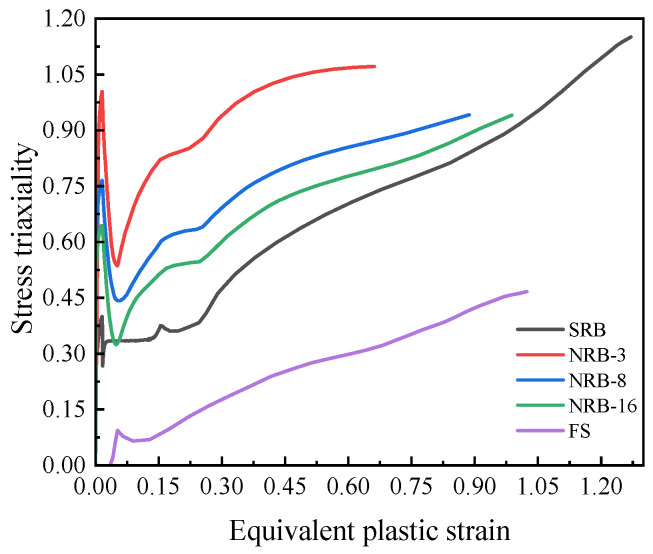
Evolution of stress triaxiality and equivalent plastic strain.

To extract fracture parameters, the complete deformation history of the critical element (i.e., the first element to fail in the model) needs to be obtained from the FEM simulation; this element is the crack initiation point, the first in the entire specimen to reach the fracture criterion. Therefore, the fracture strain refers to the equivalent plastic strain accumulated in this critical element when the applied displacement reaches the experimental fracture point. Using the full deformation history, the variation in stress triaxiality with equivalent plastic strain was determined and is presented in Figure 12. The endpoint of this curve represents the material’s fracture initiation point. Since fracture is the cumulative result of the entire plastic deformation history, the average stress triaxiality is calculated by the integration method (3).

As shown in Figure 12, these evolution curves all exhibit nonlinearity. The value of η is not monotonically increasing: in the initial stage of loading, η increases sharply, then a clear drop occurs, and finally it rises continuously with the accumulation of plastic strain. The experiment quantitatively verified that geometric constraint (notch radius) has a decisive influence on the stress state: the smaller the notch radius (i.e., the stronger the constraint), the higher the increase and maximum value of stress triaxiality.

As the average stress triaxiality increases from about 0.23 to about 0.92, the fracture strain drops from 0.913 to 0.662 (approximately a 28% decrease), indicating the strong detrimental correlation between stress state and ductility.

In addition, the average stress triaxiality of the shear specimens (FS) significantly deviated from the theoretical value of 0. This is because the specimens retained the original curvature of the pipe, causing them to be in a state of not pure shear during loading, but rather subjected to the combined effect of tensile stress. FS specimens retained the original curvature to avoid work-hardening induced by flattening. The resulting deviation from pure shear was fully captured by the FE model, yielding a calibrated triaxiality of 0.23.

### 3.4. Fitting of the Failure Model

The Johnson–Cook failure model [25] can well predict the fracture behavior of metals under high stress triaxiality; it expresses fracture strain as an exponential function of stress triaxiality:

(11) $${\bar{\varepsilon }}_{f}=\left[d_{1}+d_{2}\exp(d_{3}\eta )\right]\left[1+d_{4}\ln(\frac{{\dot{\bar{\varepsilon }}}^{p}}{{\dot{\varepsilon }}_{0}})\right](1+d_{5}{\overset{⌢}{\theta }}^{m})$$

where $1+d_{5}{\overset{⌢}{\theta }}^{m}$ is a temperature-related term, and $d_{1}-d_{5}$ are parameters determined by experiments.

Given that the experiments were performed under quasi-static conditions and temperature changes during loading were insignificant, the temperature- and strain-rate-dependent terms in Equation (11) were omitted, leading to a simplified form:

(12) $${\bar{\varepsilon }}_{f}^{\mathrm{pl}}=d_{1}+d_{2}\exp(d_{3}\eta )$$

**Figure 13:**
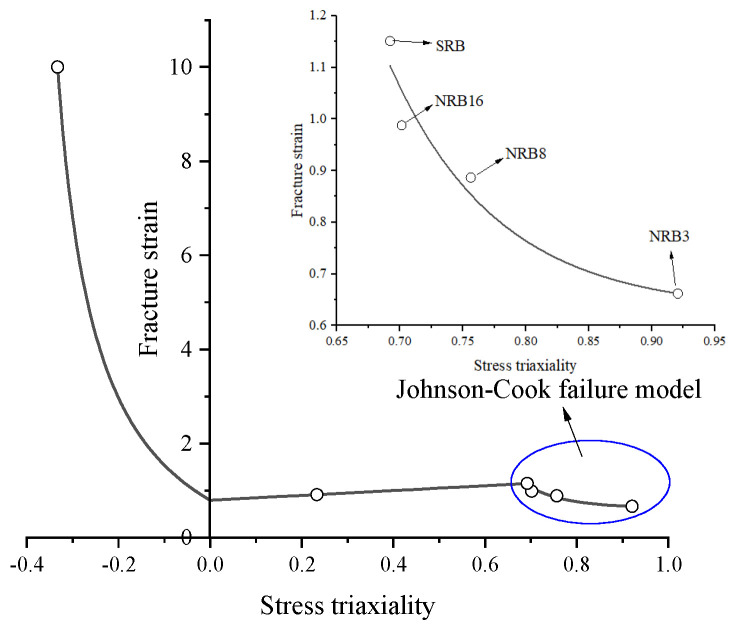
Failure model for L360QS pipeline steel.

The previously determined average stress triaxialities and fracture strains are listed in Table 3. Based on these data, the failure model function for L360QS pipeline steel was constructed. The smooth and notched tensile specimens were used to calibrate the Johnson–Cook failure model, whereas the smooth tensile and shear specimens (η between 0 and medium levels) were described by a linear relation, whose y-intercept defines the fracture strain at η = 0. Reference [57] (Bai & Wierzbicki, 2005) found a “fracture cutoff” effect for materials; i.e., the stress triaxiality approaches infinity as η approaches −1/3. Based on this, in this study the fracture strain at η = −1/3 was taken as a relatively large number (10) to represent the case that the compression specimen does not fracture. The same functional form as used in Reference [26] (Bao & Wierzbicki, 2004) was adopted to describe the fracture strain for η from −1/3 to 0, as given by (13). In summary, the constructed failure model is shown in Figure 13.

(13) $$\left\{\begin{array}{l} {\bar{\varepsilon }}_{f}=0.6289+1495.0878\exp(-11.6401\eta )(0.692<\eta <0.921)\\ {\bar{\varepsilon }}_{f}=0.51806\eta +0.79234(0<\eta <0.692)\\ {\bar{\varepsilon }}_{f}=\frac{1}{0.41924+\eta }-1.59296(-0.333<\eta <0) \end{array}\right.$$

As can be seen from Figure 13, the relationship between fracture strain and average stress triaxiality differs significantly across different triaxiality ranges. Specifically, in the range of negative stress triaxiality (η = −1/3 to 0), fracture strain decreases noticeably with higher triaxiality, following an exponential law (exponential decay trend); in the range η = 0~0.692, due to the limited number of experimental data points, a simple linear fit was used, showing a monotonically increasing trend; in the high stress triaxiality range η = 0.692~0.921, fracture strain decreases with increasing stress triaxiality, exhibiting a curving downward trend consistent with the exponential function distribution of the Johnson–Cook failure model.

### 3.5. Validation of the Failure Model

The accuracy of the proposed failure model for L360QS pipeline steel was evaluated through a train–test split validation method. First, the notched specimen experimental data were divided into a training set and a test set [44]. The training set was used to fit the failure model and determine the parameters (through optimization algorithms); then, the test set was used to evaluate the model’s accuracy by calculating error metrics, thereby verifying the model’s generalization ability. Parameters were calibrated using the Levenberg–Marquardt non-linear least squares optimization algorithm to minimize the residual sum of squares between the model predictions and experimental data. In this study, NRB3, NRB8, NRB16, SRB, and FS specimens were used as the training set, and NRB5, NRB37, and NRB113 specimens were used as the test set.

**Figure 14:**
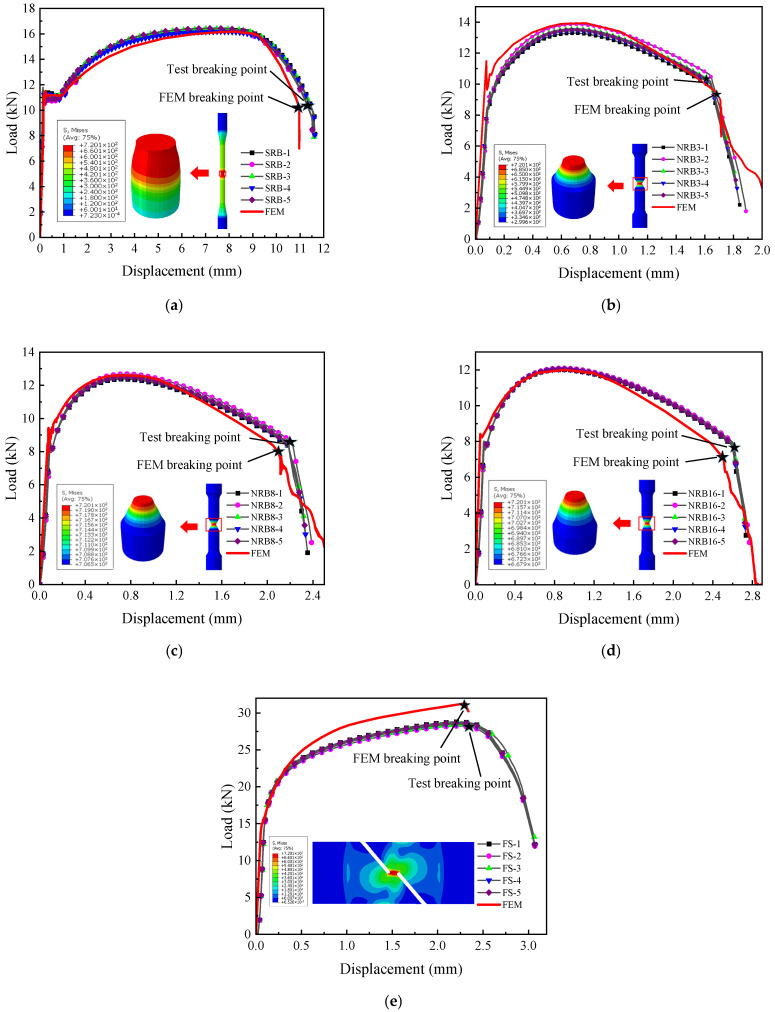
Comparison between finite element simulation and experimental fracture for training set: (**a**) SRB; (**b**) NRB3; (**c**) NRB8; (**d**) NRB16; (**e**) FS.

First, the failure model was implemented in ABAQUS finite element software to simulate the fracture process of the training set specimens, and through continuous optimization, results as shown in Figure 14 were obtained. From the comparison results, it can be seen that the fracture points in the numerical simulations match well with those in the experiments, with errors all within 5%, as listed in Table 4.

**Figure 15:**
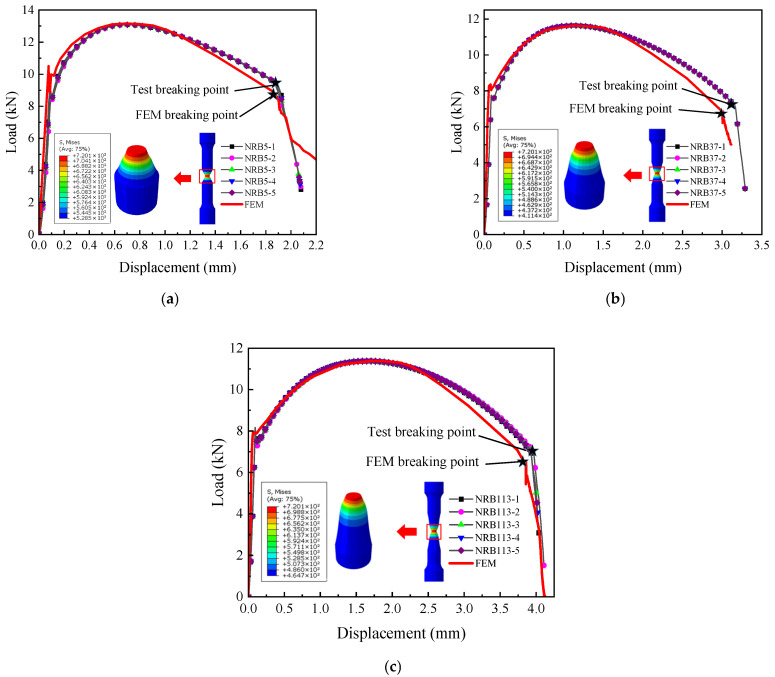
Comparison between finite element simulation and experimental fracture for test set: (**a**) NRB5; (**b**) NRB37; (**c**) NRB113.

The failure model was subsequently employed in ABAQUS to simulate the fracture response of the test set. As illustrated in Figure 15, the numerical and experimental fracture points exhibit excellent consistency, with deviations within 5%, demonstrating the precision of the proposed model.

When evaluating the model’s accuracy, minor discrepancies in the experimental data are inevitable. These deviations are primarily due to the limitation of using an uncoupled fracture model, which does not simulate the progressive stiffness degradation associated with void coalescence, as well as the neglect of plastic anisotropy inherent in rolled pipelines. The discrepancy in the FS specimen (approx. 4%) is attributed to shear localization. The continuum damage model tends to overestimate ductility in shear bands where void sheeting accelerates failure faster than void growth models predict.

Despite such experimental error, the overall congruence between the finite element simulation results and the experimental data is high. This very good agreement strongly indicates that the established L360QS true stress–strain (constitutive) model and the subsequently constructed failure model are accurate and reliable.

In the experiments of the standard tensile (SRB) specimen and all notched (NRB) specimens, it was observed that at the onset of the loss of load-bearing capacity (fracture initiation), no macroscopic crack was observed on the outer surface at the specimen’s notch. In the finite element model, the critical element always appeared at the center of the specimen. This observation is consistent with the model’s predictions and thus supports the validity of the model’s assumption regarding the fracture initiation location.

## 4. Discussion

### 4.1. Analysis of the L360QS Fracture Locus

The primary result of this study is the calibrated fracture locus (Figure 13). The most significant scientific finding from this data (Table 3) is the non-monotonic relationship between fracture strain and average stress triaxiality for L360QS steel.

The fracture strain increases from 0.9131 at $\eta_{avg}=0.2331$ (FS specimen) to a peak of 1.2707 at $\eta_{avg}=0.6920$ (SRB specimen). Only after this peak does the ductility begin to decrease sharply with increasing triaxiality, as predicted by classical void-growth models. This behavior, while seemingly counter-intuitive, is consistent with the advanced fracture mechanics framework proposed by Bao and Wierzbicki [26]. Their work identified a ductility “valley” at low triaxiality (e.g., 0<η<0.3) where shear-dominated failure mechanisms are active. Consistent with the classical Rice and Tracey theory, the failure mechanism shifts to one governed by void growth as triaxiality increases beyond this valley. In this intermediate regime, moderate hydrostatic tension (which defines η) can increase ductility by suppressing the formation of localized shear bands, allowing the material to sustain more plastic strain before void coalescence occurs.

The data suggests the toughness “peak” for this high-grade L360QS steel occurs at a relatively high triaxiality ($\eta_{avg}\approx 0.7$). The three-part piecewise model (Figure 13) presented in this work must be interpreted in this context. The exponential fit for $\eta_{avg}>0.7$ and the power-law fit for $\eta_{avg}<0.23$ are physically justified. However, the linear segment ($0.23\leq \eta_{avg}\leq 0.69$) is a pragmatic interpolation based on sparse data (only two points). While this bi-linear/tri-linear simplification is highly effective for implementation in engineering FFS software and is proven accurate as confirmed by the present validation (Table 4), the true physical locus in this region is likely a complex non-linear curve.

### 4.2. Correlation of Geometric Constraint and Stress State

#### 4.2.1. Feature Matrix Construction

To further explore the high sensitivity of L360QS pipeline steel’s fracture behavior to specimen geometry (i.e., notch radius), a correlation analysis was performed. The feature matrix (Table 5) used is based on data back-calculated from the established failure model (Figure 13). Specifically, the geometry and initial stress state of all 7 specimens (training set: SRB, NRB3, NRB8, NRB16; and test set: NRB5, NRB37, NRB113) were input into the fitted failure model to inversely solve for an idealized set of average stress triaxialities and fracture strains. To further verify whether the strong monotonic relationships revealed by the model manifest in real material behavior, a Spearman correlation analysis [45] was also performed on the original experimental data. Using the experimental fracture displacements, an average stress triaxiality and fracture strain were inversely obtained (for each specimen), as shown in Table 6. The chosen features cover the complete chain from initial geometry to final failure.

The geometric feature uses notch curvature (1/R) as the core variable. Using curvature instead of radius is standard practice in statistics, as it allows the SRB specimen to be treated as a numerical value (1/R = 0) and makes the data comparable. The stress state features include the initial triaxiality calculated by Bridgman’s formula and the FEM-back-calculated average triaxiality. The failure feature uses the FEM-back-calculated fracture strain.

#### 4.2.2. Spearman Correlation Heatmap Analysis

In choosing a correlation coefficient, the traditional Pearson coefficient only applies to measuring linear relationships. However, from the failure model established in Chapter 3 (Figure 13), it is known that in the high triaxiality range, the relationship between $\varepsilon_{f}$ and $\eta_{avg}$ is highly nonlinear (exponential). Accordingly, this study employs the Spearman rank correlation coefficient, which measures monotonic relationships between variables without the necessity of linearity, implying that an increase in one variable corresponds to a consistent increase or decrease in the other. This fits the physical phenomena observed in this study. Its formula is [45]:

(14) $$\rho =1-\frac{6\sum d_{i}^{2}}{n(n^{2}-1)}$$

where ρ (rho) is the Spearman rank correlation coefficient; $d_{i}$ is the difference in ranks of the two variables for the *i*th sample ($d_{i}=\mathrm{rank}(X_{i})-\mathrm{rank}(Y_{i})$); $\sum d_{i}^{2}$ is the sum of the squared rank differences; n is the number of samples. Spearman’s ρ ranges from −1 to +1, where +1 represents a perfectly monotonic positive correlation and −1 represents a perfectly monotonic negative correlation.

**Figure 16:**
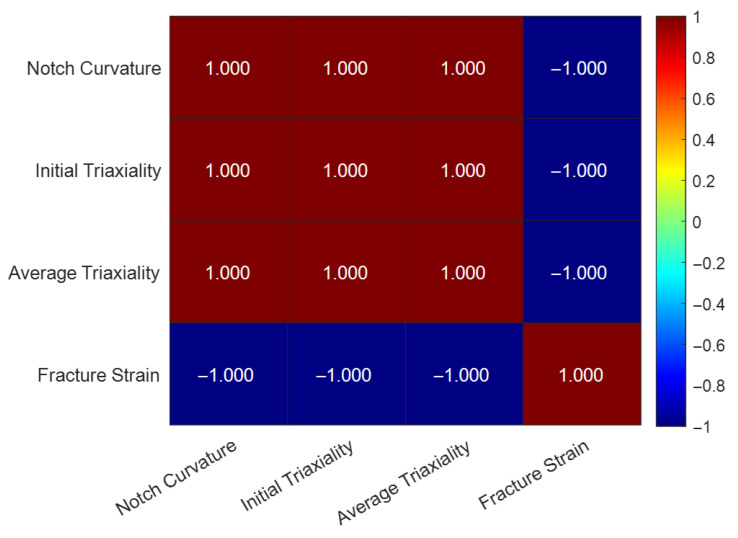
Spearman correlation heatmap for fracture behavior features of L360QS steel (model data).

Based on the model-back-calculated data in Table 5, the Spearman correlation coefficients between each feature were calculated, and the results are summarized in Table 7. Figure 16 is the Spearman correlation heatmap.

Table 7 represents ideal model predictions, resulting in perfect correlation ($\rho = 1$), confirming the model’s mathematical monotonicity. Table 8 presents the actual experimental correlations, which incorporate scatter.

**Figure 17:**
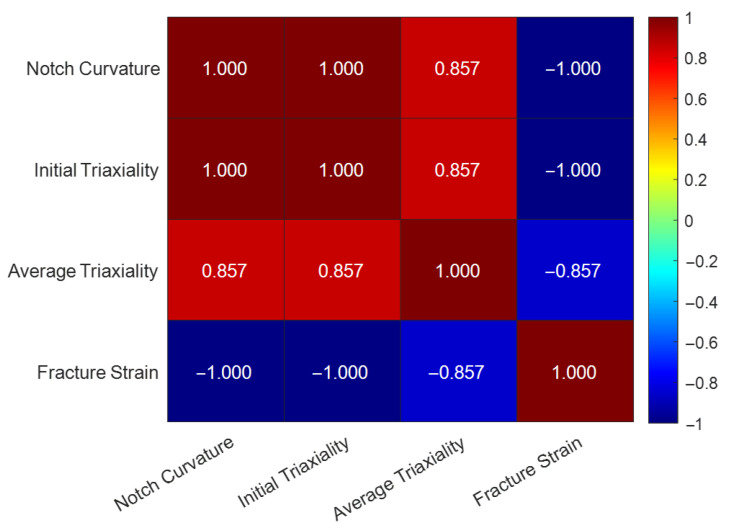
Spearman correlation heatmap for fracture behavior features of L360QS steel (experimental data).

The Spearman rank correlation analysis was performed using the experimentally determined data from Table 6, and the calculated coefficients are presented in Table 8. Figure 17 is the Spearman correlation heatmap for the experimental data.

From the above, it can be seen that notch curvature (1/R) is positively correlated with both $\eta_{init}$ and $\eta_{avg}$. The experimental data (Table 8) show a high Spearman coefficient of approximately +0.857 between notch curvature (1/R) and average triaxiality. This indicates that a smaller notch radius—corresponding to a larger curvature (1/R) and a sharper notch—leads to a higher average stress triaxiality. Average stress triaxiality is negatively correlated with fracture strain. This quantitatively confirms the strong negative correlation of stress triaxiality on fracture strain (i.e., stress triaxiality is the primary physical quantity causing a decrease in material ductility/fracture strain). The high positive correlation between $\eta_{init}$ and $\eta_{avg}$ (ρ ≈ +0.857 in experimental data) answers the question raised in Section 4.1 and corroborates the concern in Section 2.2. It indicates that although triaxiality evolves dynamically during the loading history, the average stress state of the entire loading process is largely “locked in” by the initial geometry-determined $\eta_{init}$.

At the same time, both heatmaps also show that after considering the real material’s microscopic heterogeneity and experimental error, there are still very strong positive correlations between notch curvature (1/R) and both $\eta_{init}$ and $\eta_{avg}$, and a strong negative correlation with fracture strain, verifying the correctness of the above conclusions.

## 5. Conclusions

L360QS pipeline steel’s ductile fracture behavior under multi-axial stress states was characterized through experiments and modeling. A piecewise fracture criterion was developed and validated, yielding the following conclusions:

Fracture Model Accuracy: The combined constitutive model and segmented failure model accurately predict the mechanical response and ductile fracture of L360QS steel across stress triaxialities from −1/3 to 0.92. Model predictions of fracture initiation for independent test cases were within 5% of experimental values, demonstrating high fidelity suitable for engineering applications.

Stress State Dependency: The relationship between fracture strain and stress triaxiality in L360QS is highly dependent on the triaxiality regime. In negative triaxiality (compression-dominated) to low triaxiality (shear) conditions, the steel exhibits high fracture ductility, which drops sharply as the state shifts towards tension (power-law trend). In the intermediate range (η ≈ 0 to 0.7), a simplified linear function was fitted due to sparse data points, showing a monotonically increasing trend connecting the shear-dominated and tensile-dominated responses. In high triaxiality conditions (η > ~0.7), fracture strain declines rapidly with increasing η, displaying an exponential relationship that aligns well with the Johnson–Cook formulation. Thus, the ductile failure locus of L360QS spans power-law, linear, and exponential segments corresponding to different stress states.

Notch Constraint Effects: Spearman correlation analysis quantitatively confirmed the strongly monotonic relationships between specimen geometry, stress state, and fracture behavior in the high stress triaxiality (tension-dominated) range.

In summary, this work establishes both experimentally and analytically that L360QS pipeline steel, while highly tough under uniaxial tension, is susceptible to significant reductions in ductility under multi-axial constraint.

However, this study has certain limitations that warrant further investigation. Firstly, the experiments were conducted under quasi-static loading at room temperature, without considering dynamic effects or harsh environmental factors (such as sour gas corrosion) that L360QS pipelines may encounter. Secondly, while the finite element model accurately predicts fracture initiation, it does not simulate the crack propagation process. Thirdly, the data points in the intermediate stress triaxiality range are relatively sparse. Future work will focus on (1) validating the model under varying strain rates and corrosive environments; (2) incorporating microscopic damage evolution into the model to simulate crack formation and extension; and (3) obtaining additional experimental data for intermediate stress triaxialities to refine the fracture criterion.

## Figures and Tables

**Table 1:** Specimen types and corresponding approximate initial stress triaxialities.

Specimen Name	Notch Radius (R/mm)	Initial Stress Triaxiality	Number	Quantity
Smooth round bar	R = ∞	0.33	SRB	5
Notched round bar	R = 3 mm	0.826	NRB3	5
	R = 5 mm	0.649	NRB5	5
	R = 8 mm	0.538	NRB8	5
	R = 16 mm	0.439	NRB16	5
	R = 37 mm	0.380	NRB37	5
	R = 113 mm	0.349	NRB113	5
Shear specimen	/	0	FS	5
Compression specimen	/	−0.33	CS	5

**Table 2:** Mechanical property parameters of L360QS pipeline steel.

Elastic Modulus/MPa	Poisson’s Ratio	Yield Stress/MPa	Tensile Strength/MPa
206,000	0.3	400.073	665.40

**Table 3:** Average stress triaxiality and fracture strain of each specimen.

Specimen Number	Average Stress Triaxiality	Fracture Strain
SRB	0.6920	1.2707
NRB3	0.9207	0.6620
NRB8	0.7563	0.8867
NRB16	0.7014	0.9877
CS	−0.3333	10
FS	0.2331	0.9131

**Table 4:** Simulation and experimental parameters of each specimen.

Specimen Number	Fracture Displacement/FEA	Fracture Displacement/Test	Model Error
SRB	10.942	11.2579	0.02806
NRB3	1.6826	1.6283	0.03335
NRB8	2.112	2.1691	0.02632
NRB16	2.5122	2.5755	0.02458
NRB5	1.891	1.8874	0.00191
NRB37	3.0191	3.1503	0.04164
NRB113	3.8556	3.961	0.02661
FS	2.3048	2.404	0.04126

**Table 5:** Feature matrix for correlation analysis (from model predictions) (Note: For SRB, R→∞, thus Curvature 1/R=0).

Specimen Number	Geometric Feature: R (mm)	Geometric Feature: 1/R (mm^−1^)	Stress Feature: $\eta_{init}$	Stress Feature: $\eta_{avg}$	Failure Feature: $\varepsilon_{f}$
SRB	10.942	0	0.333	0.5632	1.0559
NRB3	16	0.333	0.826	0.9309	0.7103
NRB8	8	0.125	0.538	0.7443	0.8296
NRB16	3	0.0625	0.439	0.6829	0.9124
NRB5	5	0.2	0.649	0.8251	0.756
NRB37	37	0.027	0.380	0.6443	0.9823
NRB113	113	0.00885	0.349	0.6107	0.9867

**Table 6:** Feature matrix for correlation analysis (from experimental data).

Specimen Number	Geometric Feature: R (mm)	Geometric Feature: 1/R (mm^−1^)	Stress Feature: $\eta_{init}$	Stress Feature: $\eta_{avg}$	Failure Feature: $\varepsilon_{f}$
SRB	10.942	0	0.333	0.692	1.2707
NRB3	16	0.333	0.826	0.9207	0.662
NRB8	8	0.125	0.538	0.7563	0.8867
NRB16	3	0.0625	0.439	0.7014	0.9877
NRB5	5	0.2	0.649	0.9778	0.8216
NRB37	37	0.027	0.380	0.6539	1.0219
NRB113	113	0.00885	0.349	0.6473	1.1342

**Table 7:** Spearman correlation matrix (ρ values) for fracture behavior features of L360QS steel.

Feature	1/R	$\eta_{init}$	$\eta_{avg}$	$\varepsilon_{f}$
1/R	1.000	1.000	1.000	−1.000
$\eta_{init}$	1.000	1.000	1.000	−1.000
$\eta_{avg}$	1.000	1.000	1.000	−1.000
$\varepsilon_{f}$	−1.000	−1.000	−1.000	1.000

**Table 8:** Spearman correlation matrix (ρ values) for fracture behavior features of L360QS steel (from experimental data).

Feature	1/R	$\eta_{init}$	$\eta_{avg}$	$\varepsilon_{f}$
1/R	1.000	1.000	0.857	−1.000
$\eta_{init}$	1.000	1.000	0.857	−1.000
$\eta_{avg}$	0.857	0.857	1.000	−1.000
$\varepsilon_{f}$	−1.000	−1.000	−0.857	1.000

## Data Availability

The original contributions presented in this study are included in the article. Further inquiries can be directed to the corresponding authors.
